# Darwinian Approaches for Cancer Treatment: Benefits of Mathematical Modeling

**DOI:** 10.3390/cancers13174448

**Published:** 2021-09-03

**Authors:** Sophia Belkhir, Frederic Thomas, Benjamin Roche

**Affiliations:** 1 CREEC/MIVEGEC, Université de Montpellier, CNRS, IRD, 34394 Montpellier, France; sophia.belkhir@ens-lyon.fr (S.B.); frederic.thomas2@ird.fr (F.T.); 2 École Normale Supérieure de Lyon, Département de Biologie, Lyon CEDEX 07, 69342 Lyon, France; 3 Departamento de Etología, Fauna Silvestre y Animales de Laboratorio, Facultad de Medicina Veterinaria y Zootecnia, Universidad Nacional Autónoma de México (UNAM), Ciudad de México 01030, Mexico

**Keywords:** adaptive therapy, cancer evolution, Lotka–Volterra models, agent-based models

## Abstract

**Simple Summary:**

Many cancers develop resistance and become unresponsive to traditional treatment strategies. In this review we highlight how mathematical models can aid the implementation of alternative treatment strategies that take into account the ecology and evolution of tumors in order to circumvent the emergence of resistance. We review some of the mathematical models that can be used and that have contributed to showing that Darwinian approaches for cancer treatment, like adaptive therapy, are promising anti-cancer treatment strategies.

**Abstract:**

One of the major problems of traditional anti-cancer treatments is that they lead to the emergence of treatment-resistant cells, which results in treatment failure. To avoid or delay this phenomenon, it is relevant to take into account the eco-evolutionary dynamics of tumors. Designing evolution-based treatment strategies may help overcoming the problem of drug resistance. In particular, a promising candidate is adaptive therapy, a containment strategy which adjusts treatment cycles to the evolution of the tumors in order to keep the population of treatment-resistant cells under control. Mathematical modeling is a crucial tool to understand the dynamics of cancer in response to treatments, and to make predictions about the outcomes of these treatments. In this review, we highlight the benefits of in silico modeling to design adaptive therapy strategies, and to assess whether they could effectively improve treatment outcomes. Specifically, we review how two main types of models (i.e., mathematical models based on Lotka–Volterra equations and agent-based models) have been used to model tumor dynamics in response to adaptive therapy. We give examples of the advances they permitted in the field of adaptive therapy and discuss about how these models can be integrated in experimental approaches and clinical trial design.

## 1. Introduction

Cancer is among the principal causes of death worldwide, and cancer incidence and mortality are increasing [1]. Conventional anti-cancer treatment strategies often consist in aggressively treating tumors in order to kill the maximum number of cells possible, without killing the patient. To do so, oncologists usually treat patients with the maximum tolerated dose (MTD), until the cancer is cured or stabilized [2].

A problem is that individual tumors are heterogeneous, because of variations at the genetic/epigenetic/cytogenetic levels and in the tumor environment, which supports the emergence of resistant clones [3]. The mechanisms of resistance in cancer cells are diverse. Resistance can arise due to mutations that preexist any treatment, or occur after the start of treatment [4]. Besides, non-mutational mechanisms are being increasingly recognized as drivers of treatment-resistance in cancers [5,6]. These mechanisms rely on cancer cells’ plasticity and the ability of cancer cells to change their transcriptional program and/or their epigenome to acquire a (sometimes reversible) drug-tolerant phenotype. For example, it was recently demonstrated that some tumor cells are able to enter a state similar to diapause as a means to escape chemotherapy and targeted therapies [7]. Similarly to pesticides used in agriculture or antibiotics used against bacteria, conventional anti-cancer treatments exert a selective pressure on cancer cells and select resistant clones [8]. As a result, the evolution of resistance to therapy remains a major obstacle to curing cancer [2]. It is thus becoming a necessity to develop resistance management plans to identify, anticipate, and/or manage the potential mechanisms of resistance [9].

Many studies have emphasized the importance of considering evolutionary dynamics to find strategies to overcome resistance (see [2,10,11,12,13]), as taking into account the Darwinian processes driving the eco-evolutionary dynamics of tumors gives new perspectives for treatment strategies [2]. For example, cancer can be studied through the lens of game theory, a theoretical framework used to analyze the interactions between two individuals or populations, considered as players, where both players use strategies that result in different payoffs, and dictate the outcomes of the game. The players can display different fixed strategies (classical game theory) or adapt their strategies over time (evolutionary game theory). From this perspective, cancer treatment can be seen as a leader-follower game, where the oncologist plays first and makes rational decisions, while tumor cells solely respond and adapt to the treatment. In this case, if the oncologist uses a fixed treatment strategy to which tumor cells adapt, he or she concedes leadership to tumor cells, which leads to treatment failure [9]. Hence the proposition from Gatenby et al. [14] to use a strategy termed “adaptive therapy”. This strategy consists in applying treatment in cycles that can be adjusted according to the evolution of the tumor. With this strategy, the goal is to prolong response to treatment and patient survival by keeping the population of resistant cells under control.

When designing, testing, and implementing evolution-based strategies, we cannot fully rely on experimental approaches: In vitro experiments are not sufficient to predict exactly what will happen when a treatment is applied in vivo, and the complexity of interactions and phenomena that occur in vivo is hard to understand. Besides, it is impossible to conduct too many pre-clinical or clinical studies, for obvious practical and ethical reasons. With mathematical models, the modeler controls all the conditions relative to the tumor modeled, and can extract features that are not easily revealed experimentally. For example, in the case of adaptive therapy, it is difficult to assess the actual role of competition between tumor cells in vivo, because of the potential effects of adaptive therapy on the immune system or on vasculature, which could be confounding factors [15]. Using in silico models is a relevant solution to understand the dynamics of cancer, and rethink the conventional approach for therapy.

One of the main questions that models can help answer is assessing whether treatment strategies based on Darwinian approaches, in particular adaptive therapy, are good alternatives to conventional strategies that fail due to resistance. Besides, as adaptive therapy is a relatively new anti-cancer treatment strategy, many other questions need to be addressed, such as: for which cancers and patients is adaptive therapy really beneficial? How can we optimize adaptive therapy strategies? From a modeling perspective, which parameters need to be taken into account when modeling cancer dynamics in response to adaptive therapy, and which models are relevant to use? How can we measure them experimentally to build realistic models? In addition, importantly, how can models of adaptive therapy be integrated in clinical research and application?

Different mathematical models have been used to answer such questions. Some are based on analytical resolution [16], while some depend on computational simulations ([17,18,19,20] for example), some are at the scale of the population of cancer cells (see [14,17,20]), others at the scale of individual cells [15,18,21], and there are many distinct assumptions or parameters that can be used. As it becomes increasingly clear that fighting cancer requires collaboration between researchers from different disciplines, such as evolutionary biologists, oncologists, and mathematicians, it appears necessary to review these modeling approaches and make them comprehensible to all interested actors. In this review, we present the most important models that have been used to test, design and optimize adaptive therapy schedules. Four categories of models can be considered, depending on whether the system of equations is homogeneous or non-homogeneous, and on whether they are deterministic or stochastic. In the homogeneous and deterministic case, the models are based on ordinary differential equations that in the case of adaptive therapy resemble the competitive Lotka–Volterra equations. We thereafter name them “Lotka–Volterra” models. A second type of models is stochastic differential equation models. Then for non-homogeneous cases, there are deterministic models, such as partial differential equation models, and stochastic models, including a kind of models named agent-based models (ABMs). In the current review, we only focus on the types of models that are the most widely used to model tumor dynamics in response to evolution-based treatment strategies, that is to say Lotka–Volterra models and agent-based models. First, we introduce the principle of adaptive therapy and the first mathematical models that were used to model the effect of therapy on cancer cells. Then, we present how Lotka–Volterra and agent-based models have been used to answer questions and test hypotheses about adaptive therapy. Finally, we explain how such models can be integrated in experimental approaches and clinical trial design.

## 2. Adaptive Therapy: Principle and First Models

Mathematical modeling approaches have been used in the field of cancer research for a long time, and have proven useful to study phenomena such as tumorigenesis and tumor growth, clonal evolution, microenvironmental interactions, or invasion and metastasis [22]. There are also several studies modeling the effects of treatment strategies (for example [23,24]), and the development of resistance to these strategies (for example [25,26]), in order to understand the dynamics of tumors in response to therapy and to improve treatment outcomes.

### 2.1. Identifying the Reasons for Failure of Conventional Treatment Strategies

**Figure 1:**
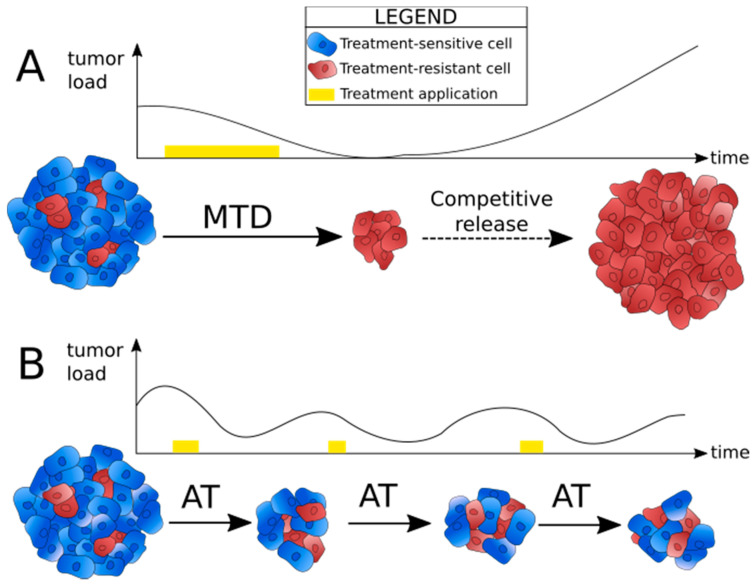
Principle of adaptive therapy. (**A**) Tumor load evolution under maximum tolerated dose (MTD) strategy. Treatment is applied continuously and at a constant dose (yellow band). Competitive release causes recurrence and treatment failure. (**B**) Tumor load evolution under adaptive therapy (AT) strategy. The on/off cycles and drug dose are adjusted to the progression of the tumor in the patient (yellow bands), and the aim is to contain the tumor at a manageable load for as many cycles as possible.

To explain why in many cases strategies based on MTD have initial success, but ultimately fail and lead to recurrence, Gatenby et Frieden mathematically modeled the effect of therapy on tumor growth [16]. To do so, they considered a tumor growth law that describes the evolution of tumor mass. This growth law contained two main terms, a free-growth factor and a limitation to growth caused by therapy. They assumed that applying a therapeutic activity modifies the growth of the tumor in a way that depends on the “level of activity” of the therapy. Their analytical resolution suggested that continuously applying a constant dose of chemotherapy destroys the cells that were the fittest before treatment, that are assumed to be the chemo-sensitive ones, but inevitably leaves behind a small number of chemo-resistant cells. These chemo-resistant cells then grow freely, because treatment is not efficient on them, and because they no longer have competitors to control their proliferation. This phenomenon is called competitive release (Figure 1A).

To face this issue, the use of time-varying therapy schedules was suggested. Metronomic chemotherapy, that follows a set schedule of on/off periods, was proposed as an alternative to continuous chemotherapy with MTD [27]. However, the results are variable. Some in silico studies suggest the use of metronomic therapy over MTD [28,29], but others showed that although metronomic therapy can prolong survival compared to treatments based on MTD, it still leads to the selection of resistant variants [14]. Besides, some pre-clinical and clinical studies, for example in advanced metastatic melanoma, showed no significant benefit of metronomic schedules over MTD [30,31]. The issue with both MTD and metronomic strategies is that they rely on a fixed schedule and dose, whereas tumors are highly labile and adaptive [32].

### 2.2. Principle of Adaptive Therapy

Since this finding, researchers have theorized and modeled new anti-cancer therapies based on Darwinian principles, meaning that they take into account the evolutionary dynamics of tumors. To date, the most proposed evolution-based strategy for the treatment of cancer is adaptive therapy, which relies on competition between sensitive and resistant cells. Indeed, within a tumor, cancer cells are in competition for resources, space, growth and survival signals from the stroma, etc. [10,11]. Treatment-resistant cells are able to escape death caused by the drugs, but their resistance mechanisms are often energetically costly. Consequently, in the absence of selection pressures created by therapy, treatment-sensitive cells are assumed to have a fitness advantage over resistant ones, and can therefore regulate the proliferation of the resistant population through cellular competition [2] (Figure 1B). Even though it may seem counter-intuitive, it means that a substantial portion of treatment-sensitive cells needs to be maintained in order to contain the tumor under a manageable volume. The optimal drug dose should thus be the minimum necessary to observe a tumor response, and once the tumor reaches an acceptable size, the drug should be withdrawn. The tumor might then grow again, but because the dominant population is composed of sensitive cells, another cycle of treatment can be started, and the drug will still have an effect [2].

Before clinically implementing adaptive therapy, the first question is to determine whether it is beneficial compared to conventional anti-cancer treatment strategies. To assess the efficacy of a therapy, different metrics can be considered, such as time to progression, time to treatment failure, or survival time [33]. Time to progression is defined as the time elapsed between treatment initiation and progression, progression being the moment at which an increase of the tumor size compared to its initial value is noticed. Time to treatment failure is the time until the tumor size reaches a threshold determined by the physician and the patient. Survival time is the time before a patients’ death, which within the scope of theoretical modeling of tumor dynamics can be defined and predicted as the time before the tumor reaches a hypothetical lethal size [33]. Predicting and comparing these metrics under different therapy regimes gives an indication on which one would be the best option. To do so, a simple way is mathematically modeling the dynamics of cancer cells in response to therapy.

The first mathematical model of adaptive therapy was developed by Gatenby et al. [14]. It is based on equations that illustrate the competition between subpopulations of cancer cells (similar to Lotka–Volterra competition equations, see Appendix A). The responses to MTD, metronomic, and adaptive therapy strategies were modeled in in silico tumors containing subpopulations of cells with different categories of resistance, associated to different fitness values. Both analytical resolution and simulations revealed that adaptive therapy could effectively permit longer survival than MTD or metronomic strategies. To complete this pioneer study, in vivo experiments on xenografted ovarian cancer treated with carboplatin confirmed the feasibility of the approach, and showed that the tumor could be controlled using progressively lower drug doses and longer off-treatment intervals [14]. This first modeling approach was a proof-of-concept that time-varying therapy schedules such as adaptive therapy can effectively be modeled and are predicted to yield better results than conventional ones. It paved the way for the development of more models aimed at designing adaptive therapy strategies, understanding the consequent tumor dynamics, and exploring the possibilities of application to various cancers (see Table 1).

## 3. Main Types of Models

The majority of models approach the question at the scale of the populations of tumor cells and are based on competition equations, that often revisit the Lotka–Volterra competition equations (see Appendix A).

### 3.1. Lotka–Volterra Models

We employ the term Lotka–Volterra for the models that use ordinary differential equations to describe interactions at the scale of the population of cells (see Appendix A). Such models have been used to model ovarian cancer [14], metastatic castrate-resistant prostate cancer (mCRPC) [17,19,44,45], breast cancer [39], multiple myeloma [38,40], colorectal cancer [47], melanoma [35,41], and lung cancer [20] dynamics in response to treatment, and showed that adaptive therapy could yield better results than conventional treatments in terms of control of the resistant populations and of survival (see Table 1).

#### 3.1.1. Identifying the Patients Who Could Benefit from Adaptive Therapy

With adaptive therapy, the goal is to contain cancer and not to cure it. This strategy is thus only proposed as an alternative in cancers that are considered incurable, in which surgery is impossible or unsuccessful and aggressive treatments will almost certainly fail, for example mCRPC [19], or in cases in which resistance appears very quickly and there are no alternative treatments, like advanced BRAF-mutant melanoma [35,41]. Using competition equations to model tumor dynamics and treatment (see Appendix A), Hansen and Read [48] tried to determine in which cases it is effectively better to treat to manage resistance instead of to attempt cure. They propose that the decision depends on the probability of cure and on the effect of a containment strategy on progression. In cases where tumors could easily be cleared by conventional strategies (surgery or chemotherapy at MTD for example), containment will not necessarily prolong survival, and therefore conventional strategies should be favored. In cases where the probability of cure is virtually null, like for metastatic and highly resistant cancers, a containment strategy such as adaptive therapy is likely to be beneficial. In intermediate situations, the best decision is ambiguous. The advantage of using mathematical models is that they allow an easy identification of the parameters that influence the position of patients on the cure-progression plane, or in other words the decision to treat with aggressive treatments or adaptive therapy. Such parameters include the expected lifespan of the patient with either strategy, the initial tumor burden, the acceptable tumor burden, the initial proportion of resistant cells, the rate of cell turnover and probability of mutation [48,49].

A crucial parameter seems to be tumor heterogeneity and the initial proportion of resistant cells. Many studies using competition equations reported that adaptive therapy is expected to be more effective when the initial proportion of resistant cells is low [17,19,39,41]. For example, in [39], simulations for different initial proportions of resistant cells suggest that adaptive therapy prolongs survival longer for tumors with a lower proportion of resistant cells. Similarly, in [19,33], both the absolute and relative gains of adaptive therapy compared to treatments based on MTD were higher when the initial proportion of resistant cells was lower. However, according to [48], adaptive therapy is more likely to be the best option for heterogeneous tumors with a large number of resistant cells. In fact, in general, any therapy is likely to have more success on tumors that have few resistant cells, but according to the real-life application considerations in [48], in these cases, we can hope for a cure, which can only be achieved with aggressive treatments. On the contrary, for heterogeneous tumors, the probability of cure is lower and that is when it would be wise to opt for a containment strategy. An essential point for implementing adaptive therapy is; thus, to be able to identify which tumors will or will not respond to it. It would be necessary to have information on the tumor composition, in particular the number, size, and drug resistance of cells from the different subpopulations, both initially and at several time-points during treatment, which is not straightforward, although techniques are being developed (see [17,38,41]).

#### 3.1.2. Designing Efficient Adaptive Therapy Schedules

Once the general categories of patients who could benefit from adaptive therapy have been identified, optimal treatment schedules have to be defined. Parameters such as the time at which treatment begins, the volume at which the tumor is contained, the threshold to start increasing/reducing drug dose or switch treatment on/off are likely to have an impact on the outcome of therapy. Models are a key tool to test and define values of these key parameters.

First, we need to know when to start treatment, in particular, once a cancer has been detected and it has been decided to treat it with adaptive therapy, is it better to start treating immediately to control the tumor, or is it better to delay until it is really necessary? A model of adaptive therapy applied to mCRPC suggested that the most efficient strategy was to delay treatment, with the aim to preserve the sensitive population and let it regulate the resistant cells for as long as possible, and apply adaptive therapy with the minimum dose possible only when really necessary [17].

Adaptive therapy cycles usually include periods off treatment or vary the drug dose. A study that modeled the response of tumors to two adaptive strategies with different on-treatment periods but equal time off concluded that the strategy with longer on-treatment periods performed better [44]. The event triggering a treatment vacation or a drug dose modulation sometimes is a change in tumor size, and it is possible to use models to estimate the volume for which switching treatment on or off would have the most impact [41]. If it is complicated to directly measure the tumor size, a change of quantity of a biomarker can serve as an indication [19,38,46]. One of the benefits of modeling strategies in silico is the possibility to implement dosing algorithms without having to know the precise doses and treatment schedules in advance. Indeed, it is easy to test different percentages of drug modulation or different thresholds for switching treatment on or off or changing the drug dose, and to select the values for which simulations predict the best outcomes. In the reference study on mCRPC [19], adaptive therapy was implemented with a treatment-stopping criterion based on the tumor burden, measured indirectly via a biomarker (prostate-specific antigen): Treatment is stopped when the tumor burden has decreased to 50% of its initial value and restarted when it has increased back to its initial size. However, it has been suggested that using other strategies and treatment-stopping criteria, like starting at a different, possibly larger, baseline burden, could further increase the benefits of adaptive therapy [50]. In some conditions, choosing a smaller tumor reduction criterion (like 20% of the initial tumor volume) may be more effective in delaying tumor progression, as illustrated with Lotka–Volterra models of advanced metastatic melanoma [35].

Another question is to decide at which size to contain the tumor. This size cannot be too small, because of the need to maintain a substantial population of sensitive cells [40]. The default strategy could be to maintain the tumor at its initial size [48], but other options can be considered, such as containing the tumor at the maximum tolerable size, or at an intermediate threshold [33]. Which option is best depends on the objective set for treatment, as containment at the initial size maximizes time to progression, while containment at the maximal tolerable size maximizes time to treatment failure [33]. There are instances where the authors used their models and data from clinical trials to estimate the minimum stable tumor volume that delays relapse [40]. In practice, when adaptive therapy is applied to patients, flexibility is allowed and tumor size is not necessarily fixed at a precise value [49,51]. However, this has to be carefully monitored, as keeping a substantial tumor volume over long periods of time or having peaks at high volume values could possibly have negative consequences for patients’ health, and lead to poor prognosis. For example, it was pointed out that many modeling studies on adaptive therapy do not consider the cumulative risk for patients of having to survive with a higher tumor burden than with conventional therapies [52].

In brief, there are many parameters to consider in order to define optimal treatment schedules, and different strategies can be designed. Identifying the best strategy depends on the objective that is set for the treatment (minimizing tumor volume, maximizing survival, maximizing time to progression, decreasing the time or cost of treatment, etc.) [20,33]. A good way to standardize optimization of schedules is to use optimal control theory. In an optimal control problem, the objective is defined beforehand, then different treatment strategies are simulated, and the best strategy is analyzed to assess what values of parameters led to the completion of the objective [34]. Many studies successfully used optimal control theory to identify the best therapy schedules, and adaptive therapy was often considered to be the best option [20,34]. The exact design of the optimal adaptive therapy strategy (length of cycles, drug dose, starting time, etc.) depends on the objective of the therapy.

Some studies have highlighted that the outcomes of the simulations were greatly dependent on the parameterization of the model. For example, Farrokhian et al. [53] pointed out that the Lotka–Volterra model they tested had a considerable sensitivity to even small alterations in the relative carrying capacities of the two subpopulations considered. In [34], it was the initial tumor composition that had a significant effect on the outcomes of treatment protocols. This is why assessing the robustness of the strategies used is important before considering a clinical application of the models. This has, for example, been done by Cunningham et al. [17]. For the relevant treatment schedules, the authors assessed whether small shifts in the administration of treatment would significantly modify patients outcomes. Most of the optimal strategies were quite robust, except for the one with the objective of minimizing the cumulative T− (resistant to abiraterone) population, which was robust for the best responder patients but not for the responder and non-responder patients. Selecting treatment strategies that are robust should thus be an important preoccupation for researchers wanting to implement models in vivo.

#### 3.1.3. Improving the Models

As of today, many models of adaptive therapy have showed that this strategy can indeed prolong survival, and have brought out the important parameters that need to be known to successfully implement this therapy. Some studies then started to focus on understanding better the assumptions underlying adaptive therapy, and on improving previous models.

One of the main assumptions in the first studies on adaptive therapy was that resistance to treatment comes with a fitness cost, meaning that in the absence of treatment, sensitive cells are fitter than resistant ones [14]. Some studies that used this assumption in their mathematical model verified the presence of a fitness cost of resistance in vitro [42,47], but it has been experimentally shown that resistance is not always costly [42], and/or that compensatory mutations decreasing the initial costs can be subsequently selected [54]. It is then reasonable to interrogate the role of fitness cost of resistance, and its necessity for adaptive therapy to be beneficial. The answers to this question are contrasted. For example, [48] showed that adaptive therapy is more likely to be a better option than aggressive treatments if resistance is considered to have a fitness cost. In a Lotka–Volterra model of adaptive therapy in colorectal cancer, it also appeared that the benefit of adaptive therapy over treatment based on MTD is negligible if there is no substantial fitness difference between sensitive and resistant cells [42]. Moreover, Silva et al. [39] highlighted the importance of the fitness cost of resistance in their model of breast cancer. They showed that adaptive therapy combined with anti-pump drugs that increase the fitness disadvantage of resistant cells prolonged survival for longer than adaptive therapy without the anti-pump drugs. Yet, Hansen et al. argued that the presence of a fitness cost does not always increase competitive suppression of resistant cells by sensitive cells, and that the nature of the fitness cost (for example whether it has an effect on resistant cells’ proliferation rate or rather on their competition ability) strongly matters [49]. Besides, Strobl et al. suggested that if there is a small level of pre-existing resistance, but a strong competition between cells, and that tumors are close to the maximal load that can be sustained by their environment, also called their environmental carrying capacity, a cost of resistance is not necessarily required [42]. They used a Lotka–Volterra model that included cell turnover as a parameter and indicated that cell turnover modulates the need for a cost of resistance. Indeed, a high cell turnover amplifies the effect of competition and therefore the benefits of adaptive therapy. In another study, Viossat et Noble simulated tumors’ response to treatment without assuming any costs of resistance, and in spite of that, adaptive therapy was still predicted to outperform MTD-based treatment strategies [43]. Once again, the nature of resistance costs matters, but overall the presence of costs of resistance tends to increase clinical gains. In particular, they showed that indefinite containment of the tumor via adaptive therapy would only be possible if sensitive cells substantially impaired the fitness of resistant cells. Additionally, some ecological phenomena and/or stochasticity events could contribute to making adaptive therapy effective, even without a resistance cost. In brief, it seems that a fitness cost of resistance is not absolutely necessary for adaptive therapy to work, but is certainly helpful [33,43].

Another aspect that can be worth considering, which was not included in the first adaptive therapy studies, is the possibility of phenotype switching between the different cell types, in particular the possibility for sensitive cells to become resistant and/or for resistant cells to become sensitive over the course of treatment. This possibility was included in some models through the use of parameters representing transition rates (see Table 1). In particular, Kim et al. [35] compared a simple Lotka–Volterra model to a model accounting for phenotype switching and showed that the benefits of adaptive therapy were predicted to be greater when phenotype switching was included in the model.

Most of the work on adaptive therapy simulates the use of a single drug, usually chemotherapy or targeted therapy. Treating tumors with multiple drugs could further improve the possibilities for adaptive therapy, and be even more beneficial [36]. Recent studies modeled the sequential application of several drugs in an adaptive therapy framework [45,46]. Fitting their model on data previously obtained in single-drug adaptive therapy clinical trials, they showed that combining multiple drugs could increase time to progression compared to conventional strategies and single-drug adaptive therapy. Further studies are needed to determine which multi-drug treatment schedules are clinically feasible, to optimize the timing of the secondary drug in the case of a primary-secondary approach, and to assess which combination of drugs would work. Some work has already been done in this direction, as a model of a two-drug adaptive therapy strategy revealed that antagonistic drugs are more efficient than synergistic drugs to control competitive release [37].

#### 3.1.4. Advantages and Limitations of Lotka–Volterra Models

Overall, Lotka–Volterra models are convenient models as they are relatively easy to conceive, parametrize, and analyze, and their asymptotic behavior can be studied, meaning that we can have an overview of the long-time outcomes of therapy [55]. However, one of their main limitations is that they are homogeneous and mostly deterministic, and usually do not account for micro-environment effects, possibility of random mutations, migration, cell turnover, or other parameters that could impact the outcomes of therapy. Some studies tried to mitigate this by introducing parameters representing micro-environment effect [47] or environmental sensitivity [14] in their competition equations, or by adding stochastic parameters such as cell turnover [42], or possibility of transition between sensitive or resistant states [35]. To truly incorporate stochasticity, an option is to transition to more complex, non-homogeneous and stochastic models. Furthermore, to be able to take into account the importance of spatial heterogeneity, it is wise to use spatial models, for example, spatial agent-based models (see Appendix B).

### 3.2. Agent-Based Models

Agent-based models (ABMs) are a powerful computational method to study the interaction between individual autonomous agents (the tumor cells) in order to understand the behavior of the whole system (the tumor). In addition to time, agent-based model simulations incorporate two other components: The agents and the environment or space in which these agents exist. It; thus, is a very relevant method for the modeling of tumors, which are biological systems where spatial organization has an important role.

#### 3.2.1. Applications of ABMs to Model Adaptive Therapy

Intratumor heterogeneity and spatial variations are important to consider when modeling tumor dynamics and treatment effects on cancer, as revealed by spatial ABMs [18,21]. You et al. [21]; thus, suggested that ABMs with continuous space are more appropriate than non-spatial models to model adaptive therapy in heterogeneous tumors, as they allow for flexibility of interaction rules, and take into account the presence of different local densities and of processes such as density and frequency-dependence. Notably, the intensity of competition for space and resources can be influenced by how the tumor is spatially organized. Bacevic et al. [47] showed that when resistant cells are spatially constrained by sensitive cells, their fitness disadvantage is further amplified. Moreover, studies based on two-dimensional on-lattice [47] or off-lattice [18] ABMs of tumor spheroids showed that tumors could be controlled longer if resistant cells were in the center of the spheroid versus on the edge. The number of locations where resistant cells emerge also seems to influence adaptive therapy outcomes [15].

Agent-based modeling is complementary to Lotka–Volterra models and can also be used to answer questions and assumptions regarding adaptive therapy. For example, with their off-lattice spatial ABM which modeled resistance as a continuous trait, Gallaher et al. demonstrated that, if some homogeneous tumors mostly composed of sensitive cells can be cured with treatments based on MTD, in heterogeneous tumors, only containment with adaptive therapy is successful [18]. The same model revealed that a high rate of spatial mixing through migration, a high rate of acquisition of resistance through mutations, as well as a high initial proportion of resistant cells are factors that lessen the efficacy of adaptive therapy strategies. To contain the tumor at a stable size and avoid competitive release, adaptive therapy would ideally include well-timed treatment vacations, but also vary the drug and its dose [14]. In practice, some strategies combine treatment modulation and dose vacation, while others only implement one of the two. To clarify which option is better, Gallaher et al. compared two adaptive therapy strategies: One that privileged treatment vacation, and one that privileged drug dose modulation. It resulted that, for invasive heterogeneous tumors, or tumors with phenotypic drift, a more vacation-oriented strategy confers better control of the tumor [18]. This is consistent with observations from Lotka–Volterra models that highlighted the benefits of well-timed treatment vacations to allow the sensitive cells to recover and to suppress the resistant population [41,44].

In order to determine the roles of the initial fraction of resistant cells, of tumor proximity to its carrying capacity, of resistance costs and of cell turnover in a spatial model, and to assess whether the results are similar between ABM and Lotka–Volterra models, Strobl et al. revisited their Lotka–Volterra model [42] into a two-dimensional on-lattice ABM [15]. This spatial model confirmed that adaptive therapy prolongs time to progression compared to MTD, even in the absence of a resistance cost. Once again, cell turnover appears to modulate the need for a resistance cost, as it increases the impact of competition between cells. Overall, the results are qualitatively similar to the ones obtained with their Lotka–Volterra model, but differ quantitatively. In terms of time to progression, the relative benefits of adaptive therapy compared to MTD are smaller in the ABM, and the presence of cell turnover and resistance costs also have less impact in the ABM. By taking space into account, ABMs model competition differently, and take into account the fact that resistant cells can be segregated in different colonies, and will not experience the same competitive inhibition depending on their neighborhood (sensitive cells that will inhibit them in an inter-specific way, or resistant cells that cause intra-specific competition). The stochastic effects present in ABMs may also alter the dynamics of tumors in response to treatment. Finally, similarly to Lotka–Volterra models, ABMs can be combined with optimal control theory to design optimal time-varying treatments [56].

#### 3.2.2. Advantages and Limitations of ABMs

In summary, agent-based modeling is an interesting approach for modeling tumor dynamics in response to adaptive therapy. Compared to Lotka–Volterra models, Agent-Based Models (ABMs) are more flexible and provide a more natural description of biological systems, in particular because they can take space into account and model the interactions between agents and between agents and their environment [55] (see Appendix B). Among the interesting features of ABMs are the fact that they can integrate multiple biological scales and recapitulate emergent behaviors, which are very relevant when studying cancer. However, ABMs are costly computationally and harder to analyze than differential models such as population-scale Lotka–Volterra models [55,57]. Indeed, due to the numerous interactions between different agents, these models can be complex to develop, an in particular, there is the issue of adequately selecting and representing the features of interest, as there are countless possibilities. Researchers may; thus, face challenges in choosing adequate model variables and testing and validating the models against experimental or clinical data [57]. Besides, ABMs embrace a variety of possible spatial representations, in particular with the possibility to consider on-lattice or off-lattice models. The choice of spatial representation in the model is not trivial either, especially considering that for now most of the clinical data is non-spatial, and yet this matter can also influence the outcomes of simulations [58].

## 4. From Models to Clinical Applications and Vice Versa

**Figure 2:**
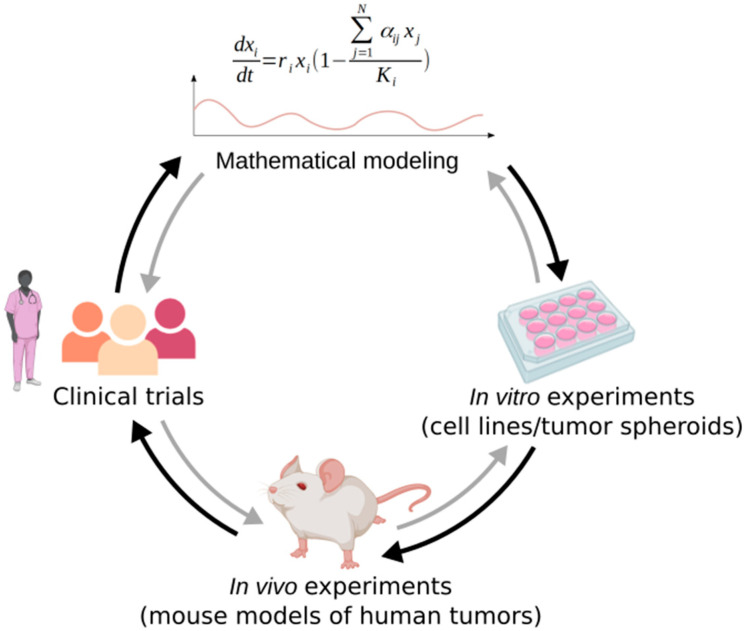
Mathematical modeling integration in clinical investigation. Mathematical models can be used to design in vitro and in vivo experiments, and clinical trials. Conversely, the retrospective analysis of experiments and clinical trials can help improve models, understand the dynamics they predict, and define the values of their parameters. Adapted from [25], created with BioRender.

Models are sometimes criticized for not considering clinical realism, and neglecting parameters that could have an importance in clinical setups. Admittedly, models are intrinsically a simplified representation of reality, as a model that is too complex would be too hard to parameterize and to understand [11]. However, they remain a powerful tool to predict and represent tumor dynamics, and elucidate important clinical parameters. Among the mathematical models presented in this review, some were validated in vivo in preclinical studies on mice [14,41,51] and a few have been successfully used to design clinical trials [19,45] (Figure 2). For example, it was shown that adaptive therapy significantly prolongs progression-free survival in different preclinical models of breast cancer [51]. Most notably, in mCRPC pilot clinical trial [19], only one out of 11 patients undergoing adaptive therapy progressed, and the 10 other maintained stable oscillations of tumor burden, while in the contemporaneous cohort treated with MTD, 14 out of 16 patients progressed. With adaptive therapy, the time to progression is estimated to be at least 27 months, and the drug dose used is considerably reduced (cumulative drug use of 47% of MTD dosing).

Conversely, the data obtained from experiments or clinical trials can be used to help models predict more realistically the tumor dynamics in response to therapy in patients (Figure 2). Indeed, as stated previously, parameterization of the models is not an easy task. To have a model that recapitulates reality as accurately as possible, it is best to use parameter values that are empirically derived. The basic parameters such as cell populations’ growth rates are often determined in vitro using cell lines with the relevant phenotypes [35]. For example, Smalley et al. calibrated their model using tumor growth dynamics from melanoma xenografts grown under vehicle, continuous, or several types of intermittent treatments with a BRAF inhibitor [41]. In the Silva et al. study on breast cancer, the growth rates of drug sensitive and drug resistant cell lines (MCF-7 and MCF-7/Dox respectively) were determined using crystal violet staining and real time quantification of the number of adherent live cells [39]. They also determined the effect of the drug on both cell lines under different metabolic conditions in vitro. In the mCRPC study by Zhang et al. [19], the values of the growth rates of T+, TP, and T− cells used in model simulations were also based on in vitro measurements of the doubling times of corresponding cell lines. In this study, the maximal carrying capacities were set arbitrarily, and the values of the competition coefficients were approximated through a series of inequalities derived from the literature and the professional judgment of oncologists. In vitro experiments are also quite useful to assess competition and fitness costs of resistance, which can be measured thanks to three methods: monoculture experiments to measure the growth rates of untreated resistant cells, co-culture of resistant and sensitive cells, and reversal experiments to explore how long it takes for resistant cells to lose their resistance mechanisms when left untreated, in a case where resistant cells can be re-sensitized [42]. The composition of a tumor in terms of number of sensitive and resistant cells and degree of resistance, which is a very useful information to have to predict the outcomes of adaptive therapy treatment, can also be assessed experimentally. For example, Smalley et al. were able to establish a link between transcriptional state composition and response to treatment and used single cell mRNA analyses to get an insight on the transcriptional heterogeneity of melanoma before, during, and after treatment [41]. To study cancer cells in a 3D configuration, it is relevant to use tumor spheroids. For instance, in [47], the spatial computational model (ABM) was used to simulate tumor spheroids, and the predictions could be compared to the experimental system. Models can also be fitted to patient data from clinical trial, to perform what is called retrospective analysis. In this case, the modelers first need to find models that fit the actual data points, and can then use these models to make predictions [15,45]. They can, for example, run simulations with different types of evolution-based strategies or treatment schedules and see what the outcomes would have been in these cases, as in [46] where they experiment with multidrug adaptive therapy approaches.

Models also help understand some phenomena observed when adaptive therapy is applied to patients in clinical trials. For example, in practice, cycle length tends to stay relatively constant for each patient, but can greatly vary between patients [19,46]. Simulations on data from prostate cancer patients reveal that the patient’s cycle length correlates with distinct spatial organizations of tumors [15]. It was; thus, proposed that the inter-patient variability could be due to differences in cell turnover and in resistance costs for drug-resistant cells, which result in different spatial distributions, and different ratios of inter-specific to intra-specific competition [15]. It also means that, knowing patient’s longitudinal response dynamics to treatment, mathematical models could be used to determine the spatial organization of the tumors.

We believe that clinical trials should systematically incorporate after-action analyses on patients who progress, to understand the causes of failure, and identify strategies that would have improved outcomes [9]. A way to do so is to use data from past clinical trials to fit mathematical models that focus on different strategies than the ones applied in the trial, so as to predict if these alternative strategies could have benefited the patients (as in [38,46]).

## 5. Conclusions and Perspectives

Reluctance to accept that treating to contain can be a better option than treating to cure, as well as the fact that there are many existing models each using slightly different approaches or parameters, can make it hard for an oncologist to be willing to implement adaptive therapy. This is why collaboration between disciplines is fundamental, and why it would be convenient to be able to clearly define when and how to use adaptive-therapy according to the situation of each patient (type of cancer, stage of the cancer, composition of the tumor, etc.), and then have a model that guides treatment decisions (when to start treatment, which drug dose to use, when to change this dose, when to interrupt and restart treatment, etc.).

We illustrated that mathematical models such as Lotka–Volterra models or ABMs are key tools to model cancer response to evolution-based therapies such as adaptive therapy. Such models allow researchers to compare adaptive therapy to other treatment strategies, identify for which cancers and which patients adaptive therapy would be beneficial, elucidate key parameters for its clinical implementation, and determine how to adjust these parameters in a patient-specific way. To simplify this review, we artificially separated models in two categories—Lotka–Volterra and ABMs—but we are aware that the reality is more complex than that and that there is a variety of models that range from analytical/deterministic models in general to more stochastic models, including spatial models such as ABMs. Of note, it is possible to combine ABMs with differential models to design hybrid models, that can also be used to describe cancer dynamics (see [22,55,59]). Overall, the models studied converge to show that adaptive therapy can prolong survival, while reducing the amount of drug used, and thus the toxicity and cost for the patient.

More research is needed to understand the dynamics of tumors in response to therapy, and the mechanisms of emergence of resistance. In particular, the mechanism of resistance emergence might influence the initial distribution of resistance cells, which can affect the response of tumors to treatment [15]. It would also be useful to have a clearer insight on the origin of tumor heterogeneity, for example through studies of the roles and organization of cancer stem cells during tumor growth [60]. Besides, the tumor environment is a complex ecosystem, where cells are in interaction with healthy cells, with other tumor cells, with the immune system, or even with the microbiota [10,61]. The nature of the competition and of the interactions between cell types also needs to be better understood. For example, for a while, adaptive therapy was thought to rely mostly on inter-specific competition between sensitive and resistance cells, but recent work identified intra-specific competition between resistant cells as a very important factor for adaptive therapy, which should be considered more thoroughly [15]. A better understanding of cell competition mechanisms, in particular between cancer cells and normal cells could also help designing therapeutic strategies that exploit competition in order to favor normal cells [61]. Apprehending and quantifying these mechanisms of competition more accurately should help refining the models, and contribute to the reproducibility of the promising results seen with adaptive therapy [49].

One of the challenges that comes with mathematical modeling is that, under a common scenario, changes in assumptions can significantly influence the outcomes predicted by the models. Given the complexity of interactions and the high number of variables at play in tumors, some models are not guaranteed to make consistent quantitative predictions, and this might explain why models are not very often incorporated into clinical practice. Another aspect that must not be overlooked is the difficulty to find appropriate experimental measurements to guide model design and parameterization. For example, actually measuring the tumor burden in a non-invasive manner and frequently enough to guide treatment decision is not a trivial task for certain types of cancer, so even relatively basic parameters like tumor volume are not always easy to estimate. Additionally, competition coefficients between cell subpopulation are important parameters in Lotka–Volterra models (see Appendix A), but it is complex to actually measure these values experimentally. In vitro approaches like clonogenic assays or competition tests are highly useful in this regard, but we have to keep in mind that there can be significant discrepancies between values obtained in vitro and in vivo. Besides, modeling approaches could still be improved and completed. Notably, most models only consider the phenotypes of the cells (“sensitive” versus “resistant”) but ignore the underlying genotypic diversity. When keeping at a given level the population size of sensitive cells, whose phenotype is targeted by the drugs, the resulting tumor can have different underlying genetic compositions, which could in turn have an importance in the emergence of resistance and metastasis initiation. For this reason, it would be relevant to design models that consider the evolution of the genotype of tumor cells in addition to their phenotype.

An aspect that also needs to be explored more in depth is the possibility of combining drugs, and types of therapies. For instance, adaptive therapy could be combined with strategies that increase the advantage of sensitive cells over resistant ones, for example, the use of benign cell boosters that select chemo-sensitive cells [62] or of fake drugs (“ersatzdroges”) that increase the metabolic cost of resistance in the absence of chemotherapy [63]. Adaptive therapy is the evolutionary-inspired strategy that has been developed the most, but other types of evolution-based therapies have been suggested and modeled (for a comprehensive review, see [2]). Many of them rely on the idea to exploit trade-offs to control tumor evolution. For instance, the evolutionary double-bind strategy exploits antagonistic pleiotropy of genes conferring resistance to a drug but increased sensitivity to another [32,64,65]. Another idea is extinction therapy, which takes advantage of the vulnerabilities of small populations to maximize the probability of cure [2]. Along the same lines as adaptive therapy taking advantage of the competition between treatment-sensitive and treatment-resistant cancer cells, competition with healthy tissue could also be exploited to develop therapeutic strategies. A few recent studies propose to exploit the competition between cancer cells and healthy cells to optimize treatment [66,67]. It could also be relevant to consider other types of interactions than competition, in particular cooperation. As a matter of fact, cooperation between cancer cells is essential for cancer progression and understanding how to impair cooperation could also help design efficient evolution-based strategies [68]. The development of more models to research these aspects will undoubtedly be beneficial to understand and treat cancer.

## Figures and Tables

**Table 1:** Summary of the main studies modeling adaptive therapy strategies. AT = Adaptive therapy. mCRPC = metastatic castrate-resistant prostate cancer. ^1^ Parameters of interest that are specific to the considered article, and that are not classical. What we consider as classical parameters are parameters representing tumor size, subpopulations sizes and growth rates, competition coefficients, carrying capacities and treatment sensitivity, or their equivalent, which are common to most of the models.

Reference	Model Type	Cancer Type	Stage	Number of Cell Types	Parameters of Interest ^1^	Predicted Outcomes
Cunningham et al. (2018) [17]	Lotka–Volterra	mCRPC	Advanced	3 (TP, T+, T−)	N/A	Optimized AT strategy outperforms MTD and metronomic strategies.
Cunningham et al. (2020) [34]	Lotka–Volterra	mCRPC	Advanced	3 (TP, T+, T−)	N/A	AT can delay competitive release compared to MTD, but it provides permanent control only for a small subset of initial tumor compositions. Other treatment schedules such as dose titration could be more successful.
Gatenby et al. (2009) [14]	Lotka–Volterra	Ovarian	Early (300mm3)	5 (different fitness and resistance)	Phenotypic sensitivity of population i to therapy (σi)/Environmental sensitivity (β)	AT prolongs survival compared to MTD or metronomic strategies.
Gluzman et al. (2020) [20]	Lotka–Volterra	Lung	N/A	3 (GLY, DEF, VOP)	Constants representing the benefit per unit of acidification, the benefit from the oxygen per unit of vascularization, and the cost of production of Vascular Endothelial Growth Factor respectively.	Optimized AT strategy outperforms MTD strategies and reduces the amount of drugs used.
Kim et al. (2021) [35]	Lotka–Volterra	Melanoma	Advanced	2 (sensitive and resistant)	Phenotype switching between drug-sensitive and resistant cell types (α and β rates)	AT delays time to progression, and even more when phenotypic switching is included in the model. A smaller burden reduction criterion may also result in better outcomes.
Ma et Newton (2018) [36]	Lotka–Volterra	N/A	N/A	3 (healthy, sensitive, resistant)	N/A	Optimized AT strategy controls the tumor for longer than MTD and metronomic strategies and could indefinitely balance the subpopulations of cells.
Ma et Newton (2021) [37]	Lotka–Volterra	N/A	N/A	3 (healthy, sensitive, resistant)	Nature of multi-drug interaction (additive, synergistic, antagonistic) (e)	AT strategy delays with two drugs delays recurrence for longer when the drugs are antagonistic.
Silva et al. (2012a) [38]	Lotka–Volterra	Multiple myeloma	Advanced	2 (sensitive and resistant with varying drug resistance levels)	N/A	Some patients could benefit from AT, but not all, hence the need to classify patients and determine which tumor compositions are suitable for AT.
Silva et al. (2012b) [39]	Lotka–Volterra	Breast	Advanced	2 (sensitive PGP- and resistant PGP+)	N/A	Compared to MTD, AT increases progression-free survival, and AT combined with drugs that increase the fitness disadvantage of resistant cells yields even better results.
Silva et al. (2016) [40]	Lotka–Volterra	Multiple myeloma	Advanced	2 subpopulations with different levels of sensitivity to each of the chemotherapeutic agents or combinations.	N/A	AT could prolong control of the disease, and combining drugs under AT regimes can be even more beneficial.
Smalley et al. (2019) [41]	Lotka–Volterra	Melanoma	Advanced	Transcriptional heterogeneity resulting in different levels of resistance	Phenotype switching between drug-sensitive and resistant cell types (α and β rates)	AT leads to delayed time to resistance and better therapeutic responses than MTD and metronomic strategies (confirmed in vivo).
Strobl et al. (2021) [42]	Lotka–Volterra	mCRPC	Advanced	2 (sensitive and resistant)	Cell turnover (density-independent death rate dt)	Adaptive therapy can prolong time to progression even without a resistance cost.
Viossat et Noble (2020) [33]	Lotka–Volterra	N/A	N/A	2 (sensitive and resistant)	N/A	AT containing the tumor at its initial size maximizes time to progression, while containment at the maximal tolerable size maximizes time to treatment failure. In some cases where resistant cells are sufficiently sensitive, MTD could be superior to AT.
Viossat et Noble (2021) [43]	Lotka–Volterra	N/A	N/A	2 (sensitive and resistant)	N/A	AT is likely to be optimal in a broad range of cases, including if there is no explicit cost of resistance. Clinical gains strongly depend on competition intensity and on a few key patient-specific factors.
West et al. (2018) [44]	Lotka–Volterra	mCRPC	Advanced	3 (healthy, sensitive, resistant)	N/A	Assuming a cost of resistance for chemo-resistant cells, AT achieves better time to relapse than MTD. AT with longer treatment-on periods but equal time off perform better.
West et al. (2019a) [45]	Lotka–Volterra	mCRPC	Advanced	3 (TP, T+, T−)	Weighting term to adapt payoff matrix and carrying capacity for each drug (wi)	Multi-drug AT could delay emergence of resistance for even longer than single-drug AT.
West et al. (2019b) [46]	Lotka–Volterra	mCRPC	Advanced	4 (T+, TP, T−, and doubly resistant T−/−)	N/A	Single drug AT increases time to progression compared with conventional strategies, and primary-secondary multi-drug AT is even more beneficial.
Zhang et al. (2017) [19]	Lotka–Volterra	mCRPC	Advanced	3 (TP, T+, T−)	N/A	AT prolongs time to progression compared to MTD and metronomic strategies.
Bacevic et al. (2017) [47]	Lotka–Volterra and ABM	Colorectal	Early (first detection—1.3 cm^3^)	2 (sensitive and resistant)	Micro-environmental feedback (constants that represent how strongly the tumor stimulates and inhibits vascularization for Lotka–Volterra models, constants regarding oxygen and cyclin-dependent kinase inhibitor’s diffusion, consumption and effects on cell proliferation and/or death for ABMs)/death rate (Mi)	AT outperforms MTD in the condition that there is a fitness cost of resistance.
Gallaher et al. (2018) [18]	ABM	Breast	Early	Continuous resistance %	Phenotypic drift, migration (angle of movement and persistence time)	In heterogeneous tumors with resistant cells, AT significantly delays time to progression.
Strobl et al. (2021) [15]	ABM	mCRPC	Advanced	2 (sensitive and resistant)	Cell turnover (density-independent death rate dt)	AT is on average superior to MTD in incurable and resistant tumors. High initial tumor density and low initial resistance fractions maximize its benefits, and a cost of resistance is not necessary.

## Appendix A. Lotka–Volterra Models

Lotka–Volterra models are deterministic models based on ordinary differential equations. In Lotka–Volterra models, the dynamics of each subpopulation, and; therefore, of the whole tumor, is described by equations that represent the inter-specific competition between treatment-resistant and treatment-sensitive cells populations. These equations are often based on the Lotka–Volterra competition Equation (A1):

(A1) $$\frac{dx_{i}}{dt}=r_{i}x_{i}\left(1-\frac{\sum_{j=1}^{{}_{N}}\alpha_{ij}x_{j}}{K_{i}}\right)$$

with *x_i_* the size of subpopulation *i* at time t, *r_i_* its growth rate, *K_i_* its carrying capacity (i.e., the maximal population size that can be sustained in the tumor environment), and *α_ij_* the competition matrix containing the competition coefficients for each pair of subpopulations. For instance, in models of metastatic castrate-resistant prostate cancer (mCRPC) treated with abiraterone, three subpopulations of cells have to be considered: T+ cells, that require exogenous androgen and are thus sensitive to androgen deprivation and to abiraterone, TP (testosterone producing) cells, that express the enzyme CYP17A1 which allows them to produce testosterone, making them resistant to androgen deprivation but sensitive to abiraterone (a CYP17A1 inhibitor), and T− cells, that are androgen-independent and resistant to abiraterone [19]. The competition matrix is of this form:

**Table A1:** Example of competition matrix.

	T+	TP	T−
T+	α_11_	α_12_	α_13_
TP	α_21_	α_22_	α_23_
T−	α_31_	α_32_	α_33_

The effect of therapy can be modeled as a decrease of the carrying capacity of sensitive cells [19], a decrease of growth rates [33], or be introduced as a supplementary parameter [50].

## Appendix B. Agent-Based Models

Agent-based models (ABMs) are computational models that focus on individual autonomous agents to understand the dynamics at the whole system level. In the case of cancer modeling, these agents are cells that can grow, migrate, mutate, divide, and die. These agents interact with their environment and the neighboring agents, from which they receive signals and inputs, and to which they provide outputs [57]. The behavior of each cell (its probability of surviving, of producing a daughter cell of the same type or of a different type, etc.) is dictated by rules, mostly according to probabilistic laws. For example, cell migration can be modeled by allowing cells to move in a persistent random walk, and phenotypic drift by allowing daughter cells to inherit a different proliferation rate upon division [18].

Cells can be allowed to continuously move in space (off-lattice ABMs) or restricted to a lattice (on-lattice ABMs). In either case, ABMs provide information on the spatial structure of the tumor, as in [18], where simulations from an off-lattice ABMs give an overview of the evolution of spatial structure of the tumor (size of the tumor, localization and level of resistance of tumor cells), as well as of the number of cells, in response to treatment based on maximal tolerated dose (MTD) or adaptive therapy.
